# Role of the V1G1 subunit of V-ATPase in breast cancer cell migration

**DOI:** 10.1038/s41598-021-84222-9

**Published:** 2021-02-25

**Authors:** Maria De Luca, Roberta Romano, Cecilia Bucci

**Affiliations:** Department of Biological and Environmental Sciences and Technologies, University of Salento, Via Provinciale Lecce-Monteroni n. 165, 73100 Lecce, Italy

**Keywords:** Cancer, Breast cancer

## Abstract

V-ATPase is a large multi-subunit complex that regulates acidity of intracellular compartments and of extracellular environment. V-ATPase consists of several subunits that drive specific regulatory mechanisms. The V1G1 subunit, a component of the peripheral stalk of the pump, controls localization and activation of the pump on late endosomes and lysosomes by interacting with RILP and RAB7. Deregulation of some subunits of the pump has been related to tumor invasion and metastasis formation in breast cancer. We observed a decrease of V1G1 and RAB7 in highly invasive breast cancer cells, suggesting a key role of these proteins in controlling cancer progression. Moreover, in MDA-MB-231 cells, modulation of V1G1 affected cell migration and matrix metalloproteinase activation in vitro, processes important for tumor formation and dissemination. In these cells, characterized by high expression of EGFR, we demonstrated that V1G1 modulates EGFR stability and the EGFR downstream signaling pathways that control several factors required for cell motility, among which RAC1 and cofilin. In addition, we showed a key role of V1G1 in the biogenesis of endosomes and lysosomes. Altogether, our data describe a new molecular mechanism, controlled by V1G1, required for cell motility and that promotes breast cancer tumorigenesis.

## Introduction

Vacuolar H^+^-ATPase (V-ATPase) is a multi-subunit complex that mediates transport of protons across intracellular and plasma membranes via an ATP-dependent mechanism^1–3^. V-ATPase is composed of several subunits organized in two domains, the V1 cytosolic and the V0 membrane-integral. Each subunit drives specific mechanisms that control activation and regulation of the pump. The V1G1 subunit of the pump is a component of the peripheral stalk of the V-ATPase required for efficient rotational catalysis^3,4^. The functional interaction of V1G1 with RILP (RAB-interacting lysosomal protein) and RAB7A (RAs-related in Brain 7a), hereafter referred to as RAB7, regulators of late endocytic traffic^5,6^, controls localization and activation of the pump on late endosomes and lysosomes^7–9^. Notably, acidification triggered by V-ATPase of endosomes, lysosomes, and secretory vesicles is required for vesicular trafficking, endocytosis, autophagy, receptor recycling, and protein degradation^2,3^.

Alterations of gene expression or subcellular localization of specific V-ATPase subunits are observed in malignant cells versus normal counterparts^1,2^. Deregulation of V-ATPase subunits induces defects in the activation of the pump that affect acidification of intracellular compartments and extracellular environment and correlate with cancer development, progression and metastasis formation^1,2,10^. In particular, in breast cancer there is a strong correlation between elevated expression of some subunits and advanced stages or metastasis, which are associated with aberrant V-ATPase plasma membrane localization^1,2^. Therefore, V-ATPase activity is considered an attractive target for cancer drug therapy. In fact, impaired V-ATPase activity affects the migratory ability of highly metastatic cells^2,11–15^ and metastasis formation and tumor volume in mouse animal models^16,17^. V-ATPase dysregulation is also associated to therapy resistance^2^.

V-ATPase could promote invasion and migration of cancer cells by modulating pH of the lumen of intracellular organelles required for the activation of lysosomal proteases important for the regulation of key cell signaling pathways such as the epidermal growth factor receptor (EGFR) signaling cascade that, through the activation of the GTPase RAC1 (Ras-related C3 botulinum toxin substrate 1), controls cell motility^18–20^. EGFR signaling is tightly regulated by compartmentalization and trafficking of the EGFR along the endocytic pathway where the reversible assembly of V_1_V_O_ domains is a regulatory mechanism that controls pH of the lumen of lysosomes required for receptor degradation^2,18,20–24^. This process is frequently deregulated in cancer cells and induces inappropriate activation of the EGFR that drives tumorigenesis^21,25^. In addition, increased expression and/or gene amplification of EGFR have been observed in many human cancers, including triple negative breast cancer MDA-MB-231 cells, and has been associated to increased cell proliferation and motility, disease progression and poor prognosis^25,26^.

The aberrant EGFR activation controls cell migration, at least in part, through RAC1, which is overexpressed in human breast cancer cells^24,27^. RAC1, interacting with specific factors, such as cofilin, leads to cytoskeletal reorganization, lamellipodium formation at the leading edge, focal adhesion complex formation events crucial in the metastatic cascade^19,28–31^.

Cell motility is also controlled by the trafficking of several factors to the surface of the cell or to the extracellular environment. In cancer cells, V-ATPase decreases extracellular pH and increases secretion and activation of extracellular proteases, such as cathepsins and matrix metalloproteinases (MMPs) that lead to extracellular matrix degradation facilitating migration of cancer cells^2,32–36^*.* Furthermore, overexpression of MMP-2 and MMP-9 correlates with an aggressive malignant phenotype and poor outcome in breast cancer^34,37^.

Given the importance of the V-ATPase in breast cancer, in this paper we characterized the molecular mechanism through which the V1G1 subunit of the pump controls the invasive phenotype of breast cancer cells with a particular focus on the EGFR signaling using the MDA-MB-231 model system. We showed that V1G1 negatively regulates cell motility in vitro and matrix metalloproteinases activation in vitro probably modulating EGFR signaling.

## Results

### V1G1 modulates invasive phenotype of breast cancer cells

**Figure 1:**
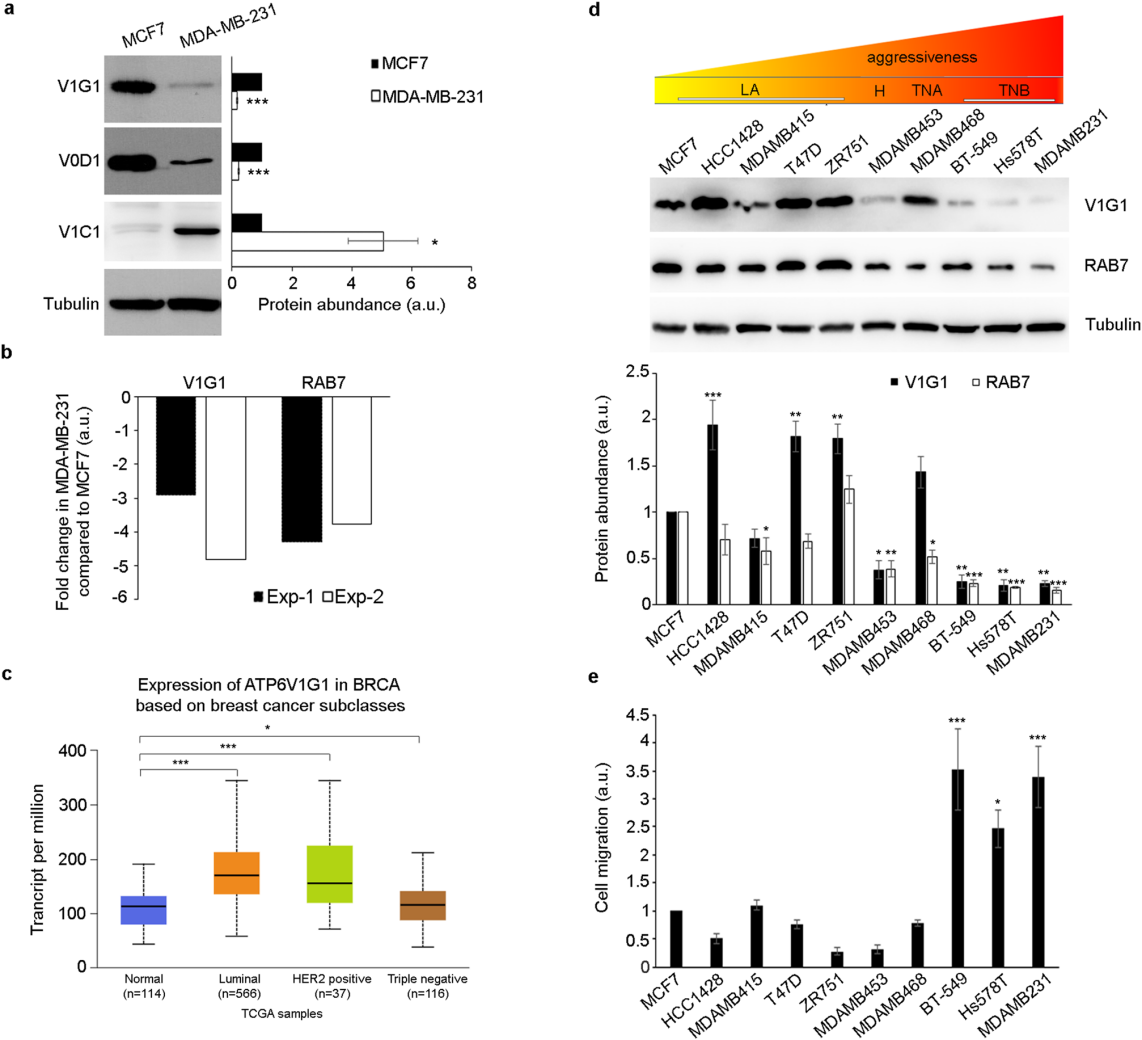
*ATP6V1G1* gene expression in breast cancer cell lines. (**a**) Lysates of MCF7 and MDA-MB-231 cells analyzed by Western blot using specific anti-V1G1, anti-V0D1, anti-V1C1 and anti-tubulin antibodies. Data represent the mean ± s.e.m. of at least three experiments and statistical analysis was performed using Student’s t-test with MCF7 as referring sample. * = *p* < 0.05; *** = *p* < 0.001. (**b**) The amount of *ATP6V1G1* and *RAB7* transcripts was quantified, compared to the *GAPDH* transcript as control, using real-time PCR. The results of two independent experiments are shown (Exp-1 and Exp-2). (**c**) V1G1 expression data from the UALCAN database in breast cancer subclasses compared to normal breast tissues. *p ≤ 0.05; ***p ≤ 0.001. (**d**) Lysates of the indicated breast cancer cell lines, belonging to LA (luminal A), H (HER2 positive), TNA (triple negative A) and TNB (triple negative B) groups, were analyzed by Western blot using specific anti-V1G1, anti-RAB7, and anti-tubulin antibodies. (**e**) Wound healing assay of different breast cancer cell lines. Cells were imaged at the initial time point (T0) and 24 h after the scratch. Cell migration was measured as the ratio between closed area of the wound in MCF7 cells (referring sample set to 1) and each cell line. Data in panels d and e represent the mean ± s.e.m of at least three experiments and statistical analysis was performed using one-way ANOVA followed by Dunnett’s multiple comparisons test with control (MCF7 cells) as referring sample set to 1 (a.u. arbitrary unit). *p ≤ 0.05, **p ≤ 0.01, ***p ≤ 0.001.

Alterations of V-ATPase subunits have been implicated in the invasive phenotype of breast cancer cells^1,2,36^. Therefore, we analyzed the abundance of some subunits of the pump in MCF7, poorly invasive breast cancer cells, and MDA-MB-231, highly invasive breast cancer cells. Western blot analysis showed an increase in V1C1 abundance in the MDA-MB-231 cell line as previously reported^38^, while we observed a decrease of V1G1 and V0D1 levels (Fig. 1a) associated to a decrease of the V1G1 transcript (Fig. 1b). These data suggest that V1G1 levels could be related to the aggressiveness of breast cancer cells. The analysis of V1G1 expression from UALCAN platform^39^ showed that while in less aggressive breast cancer subclasses V1G1 transcript levels are higher compared to normal breast tissues, in triple negative breast cancer they are reduced (Fig. 1c). Regarding protein levels, with the help of The Human Protein Atlas database^40^, we found that they are decreased in breast cancer compared to normal tissues (Supplementary Fig. 1 online). Therefore, we decided to analyze V1G1 protein levels in additional breast cancer cell lines of different subtypes: the luminal A (LA) subtype (MCF7, HCC1428, MD-MB-415, T47D and ZR75-1 cells), the HER2 positive (H) subtype (MDA-MB-453 cells), the triple negative A (TNA) subtype (MDA-MB-468 cells) and the triple negative B (TNB) subtype (MDA-MB-231, BT-549 and Hs578T cells)^41–43^. Western blot analysis showed a strong decrease of V1G1 protein abundance in TNB cell lines compared to other breast cancer cell lines of different subtypes (Fig. 1d). As V1G1 is a subunit of the proton pump that controls acidification of extracellular space required for cell migration, we analyzed cell migration in vitro using a wound healing assay on confluent monolayers of these different breast cancer cell lines. Cells were scratched and cells migrating toward the wound were imaged at T0 and 24 h after the scratch. We observed a strong increase in cell migration in TNB cell lines (BT-549, Hs578T and MDA-MB-231 cell lines) (Fig. 1e), as expected, associated to a strong decrease of V1G1 protein abundance compared to MCF7 cells (Fig. 1d), while the increase of V1G1 levels seems to be associated with a decrease in cell migration in HCC1428, T47D, ZR75-1 and MDA-MB-468 cells compared to MCF7 cells, although this reduction is not statistically significant (Fig. 1d, e).

**Figure 2:**
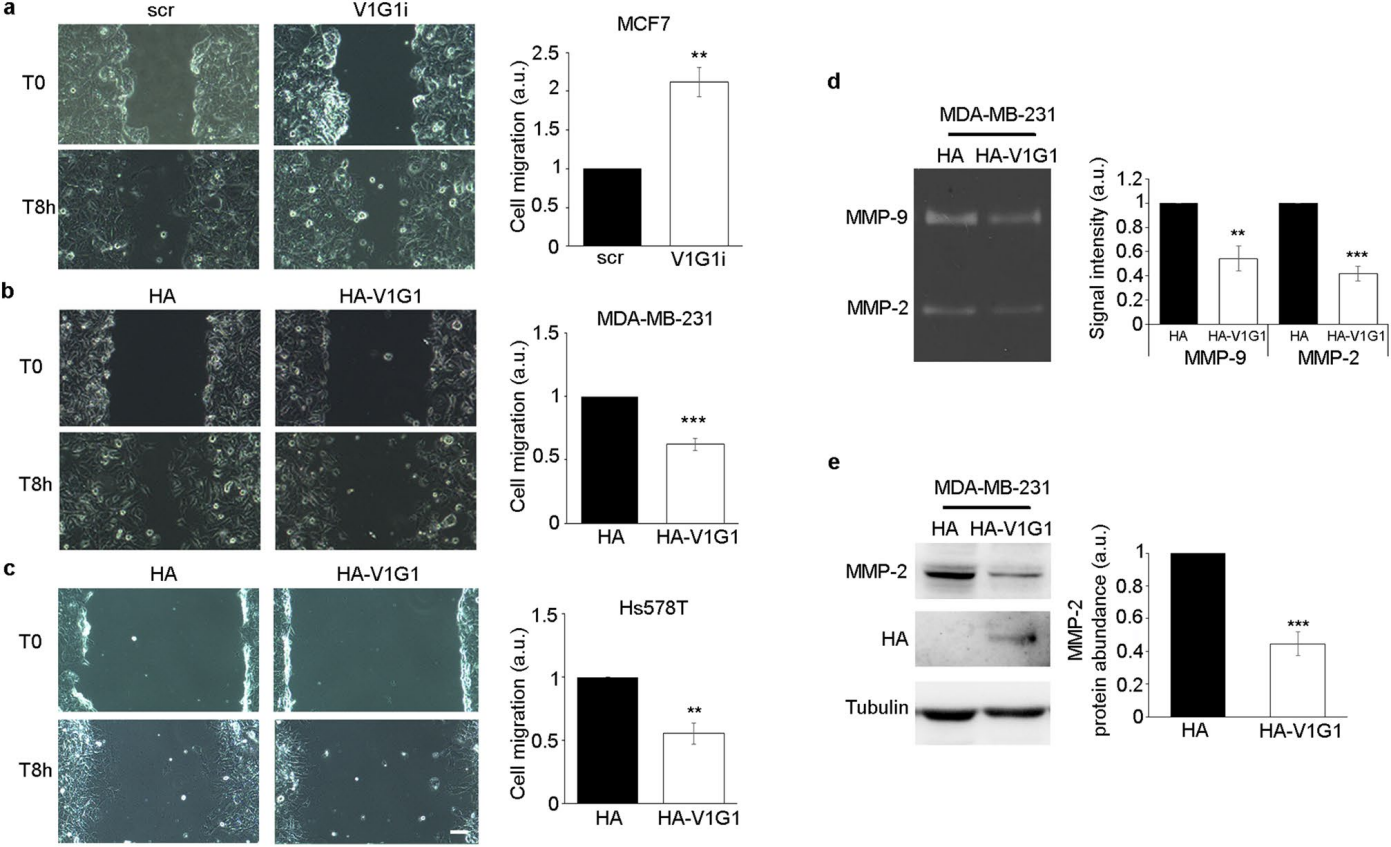
V1G1 modulates invasive phenotype of breast cancer cells. V1G1-depleted MCF7 (V1G1i) (**a**) and HA-V1G1 overexpressing MDA-MB-231 (**b**) or Hs578T cells (**c**) were imaged during wound healing assay at initial time point (T0) and 8 h after the scratch. Cell migration was measured as the ratio between the closed area of the wound in control cells (HA or scr) and HA-V1G1 overexpressing or V1G1 silenced cells. Scale bar = 100 µm. (**d**) Gelatin zymography was performed using conditioned medium of MDA-MB-231 cells overexpressing HA-V1G1 and control. Representative results are shown. (**e**) Western blot analysis was performed using specific anti-MMP-2, anti-HA and anti-tubulin antibodies. Intensity of bands was measured using the software Image Lab. Data represent the mean ± s.e.m. of at least three experiments. Statistical analysis was performed using Student’s t-test with control cells (scr or HA) as referring sample set to 1. **p ≤ 0.01; ***p ≤ 0.001.

Then, we modulated V1G1 expression in breast cancer cell lines to analyze cell migration and matrix metalloproteinases activation, two processes controlled by V-ATPase during tumor formation and dissemination^2,10^. We performed a wound-healing assay on confluent monolayers of V1G1-silenced MCF7 cells and HA-V1G1 overexpressing MDA-MB-231 cells. In wound-healing migration assay, cells migrating toward the wound were imaged at T0 and 8 h after scratch. Interestingly, we showed a correlation between V1G1 levels and cell migration. In fact, V1G1 depletion in MCF7 cells increased cell migration (Fig. 2a), while HA-V1G1 overexpression in MDA-MB-231 cells decreased cell migration (Fig. 2b). Interestingly, overexpression of V1G1 impaired cell migration in vitro also in Hs578T cells, an additional TNB cell line (Fig. 2c).

The activation of MMPs, and therefore the degradation of the molecular components of extracellular matrix, improves cell migration in vivo. A class of MMPs are the gelatinases that play a critical role in cancer progression and among which are included MMP-2 and MMP-9^37^. In order to measure the relative amounts of active enzymes secreted by MDA-MB-231 overexpressing HA-V1G1, we performed a gelatin zymography^44^. Gelatin zymography showed two strong bands for MDA-MB-231 cells, corresponding to the zone where the gelatin was digested by active MMP-2 and MMP-9, that became smaller when HA-V1G1 was overexpressed (Fig. 2d) suggesting a decrease in the activation of the proteinases in MDA-MB-231 after HA-V1G1 overexpression (Fig. 2d). In addition, we observed a decrease of the total amount of MMP-2 in MDA-MB231 cells overexpressing HA-V1G1 (Fig. 2e). These data suggest that overexpression of V1G1 affects abundance and activation of MMPs.

### V1G1 modulates membrane receptor stability and signaling

**Figure 3:**
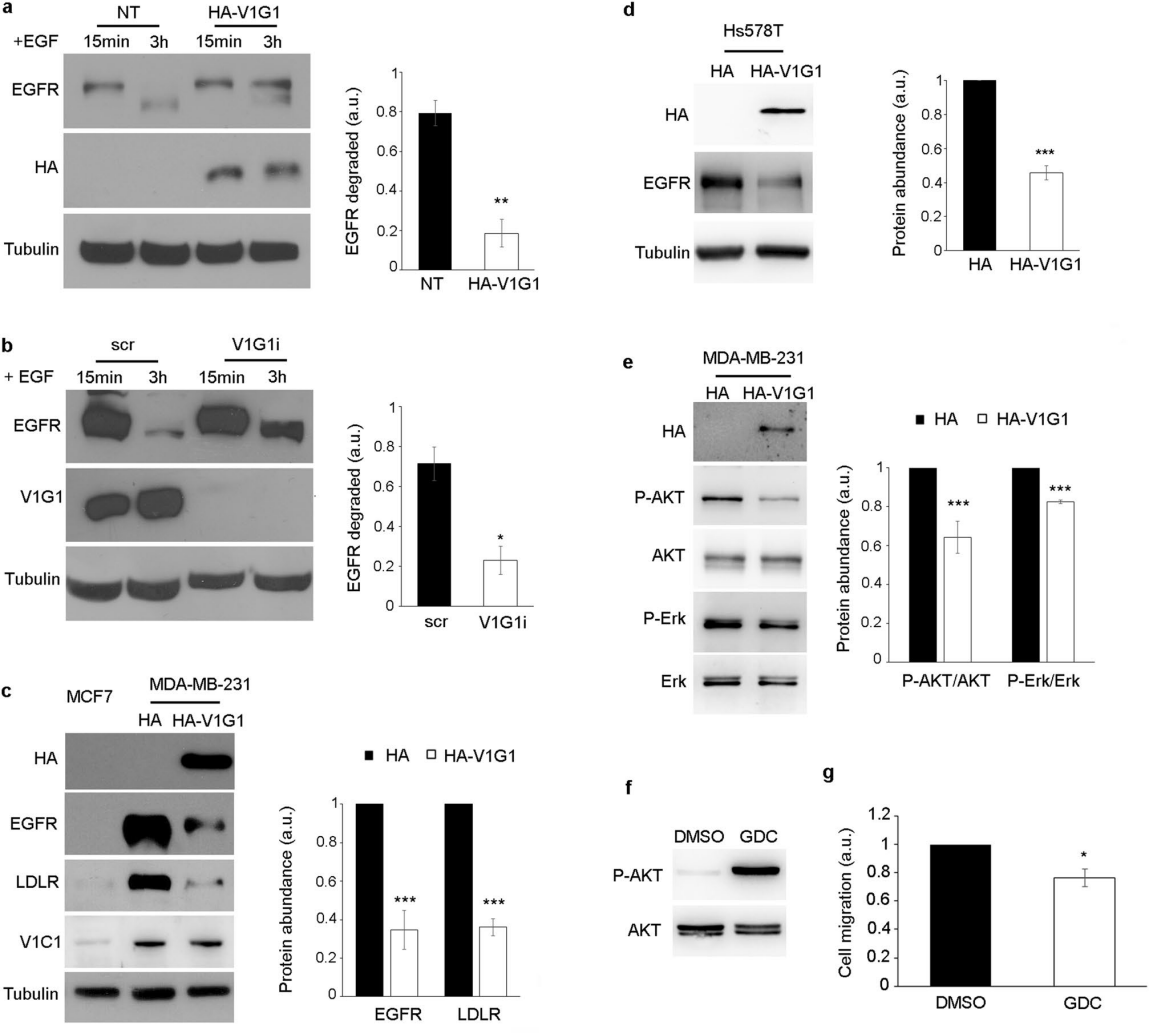
V1G1 controls EGFR stability and signaling. HA-V1G1 overexpressing (**a**) or V1G1-depleted (**b**) HeLa cells were treated with 10 ug/ml of cycloheximide for 1 h and stimulated for 15 min or 3 h with 100 ng/ml EGF. Lysates were analyzed by Western blot using specific anti-EGFR, anti-HA, anti-V1G1 and anti-tubulin antibodies. The amount of EGFR degraded at 3 h was quantified using ImageJ software and plotted as a percentage of the respective intensity at 15 min. Values at 15 min were set to 1. (**c**) Lysates of MDA-MB-231 overexpressing HA-V1G1 and MCF7 cells were analyzed by Western blot using specific anti-HA, anti-EGFR, anti-LDLR, anti-V1C1, and anti-tubulin antibodies. Intensities of bands were measured by densitometry and normalized against tubulin. (**d**) Lysates of Hs578T cells overexpressing HA or HA-V1G1 were analyzed by Western blot using the indicated antibodies. Intensities of bands were measured by densitometry and normalized against tubulin. (**e**) Lysates of control (HA) and HA-V1G1 overexpressing MDA-MB-231 cells were analyzed by immunoblotting using the indicated antibodies. Bands were quantified by densitometry and normalized against total protein. (**f**) MDA-MB-231 cells treated for 24 h with 1 µM GDC-0068 were lysed and analyzed by Western blot using the indicated antibodies. (**g**) Cell migration was measured as the ratio between closed area of the wound in control cells incubated with DMSO (set to 1) and cells treated with GDC-0068. Data represent the mean ± s.e.m. of at least three experiments. Student’s t-test. *p ≤ 0.05, **p ≤ 0.01, ***p ≤ 0.001.

Breast cancer is a highly heterogeneous tumor, as breast cancer cell lines exhibit diverse gene expression profiling and clinical features^26,41^. MDA-MB-231 triple negative cells are featured also by high levels of EGFR expression compared to luminal A cell lines such as MCF7^26^. EGFR signaling regulates cell proliferation and survival, and the overactivation of the receptor promotes cancer development^22,25^. Endocytosis plays an important role in EGFR regulation. The internalization and the subsequent degradation into lysosomes control the activation of the receptor^21,22^. In order to establish if V1G1 modulates EGFR turnover, we measured EGFR degradation in HeLa cells overexpressing and silencing V1G1. To this purpose, cells were starved, incubated with cycloheximide to prevent synthesis of new EGFR and incubated with EGF to induce EGFR degradation. The amount of EGFR was detected by Western blot analysis. The analysis showed that about 80% of EGFR, in control cells, was degraded 3 h after the incubation with EGF, while V1G1 overexpression and silencing inhibited EGF-induced EGFR degradation (Fig. 3a, b), suggesting a critical role of V1G1 in the modulation of the receptor.

MDA-MB-231 cells exhibit high expression of EGFR compared to the MCF7 cell line^26^. Therefore, to establish whether V1G1 affects EGFR in MDA-MB-231 cell lines, we overexpressed HA-V1G1 in these cells, where we observed lower level of V1G1 compared to MCF7 (Fig. 1a, d). Western Blot analysis showed a significant reduction of EGFR protein amount in HA-V1G1 overexpressing MDA-MB-231 cells (Fig. 3c) associated also to a strong reduction of Low-Density Lipoprotein Receptor (LDLR) (Fig. 3c). Interestingly, also in Hs578T cells HA-V1G1 overexpression caused a reduction of EGFR protein amount (Fig. 3d). In addition, we showed that V1C1 levels are not affected by V1G1 overexpression in MDA-MB231 cells (Fig. 3c).

EGFR stimulates several signaling pathways, such as phosphatidylinositol 3 kinase (PI3K/AKT) and Ras-mitogen-activated protein kinase (MAPK/ERK) pathways, which promote cell survival and proliferation^45^. In order to evaluate the receptor signaling, we analyzed the activation of AKT and ERK in HA-V1G1 overexpressing MDA-MB-231 cells (Fig. 3e). We observed in these cells a significant reduction in the activation of both AKT and ERK while the total amount of these kinases was unchanged (Fig. 3e). Therefore, these data suggest that V1G1, regulating EGFR signaling, could control tumor cell survival and progression. To support this hypothesis, we inactivated AKT using the GDC-0068 inhibitor (1 µM) for 24 h, thus inhibiting EGFR signaling pathway (Fig. 3f). Interestingly, inactivation of AKT induced a decrease of cell migration in MDA-MB-231 cells 8 h after the scratch (Fig. 3g).

### V1G1 affects cell migration through RAC1 dependent pathway

**Figure 4:**
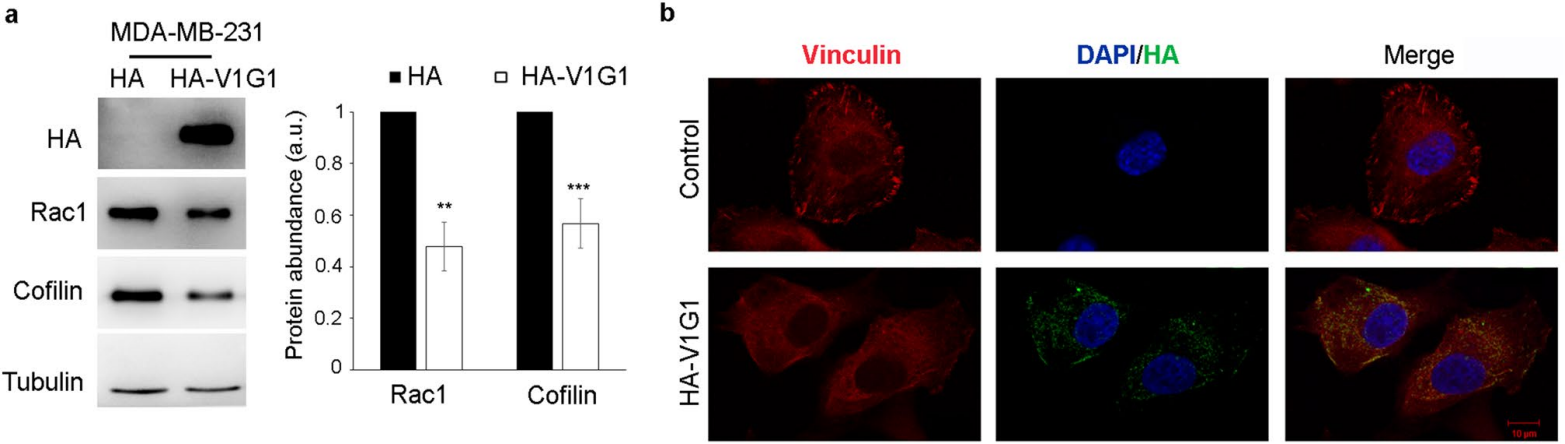
V1G1 overexpression affects RAC1 signaling. (**a**) MDA-MB-231 cells overexpressing HA-V1G1 and control (HA) were lysed and subjected to western blot analysis using anti-HA, anti-RAC1, anti-cofilin and anti-tubulin antibody. Quantification of RAC1 and cofilin protein amount is shown. Data represent the mean ± s.e.m. of at least three experiments. **p ≤ 0.01; i***p ≤ 0.001. (**b**) HA-V1G1 overexpressing MDA-MB-231 cells were immunolabeled with anti-HA followed by Alexa488 conjugated secondary antibody to discriminate between cells overexpressing HA-V1G1 and control cells, anti-vinculin followed by Alexa568 conjugated secondary antibody while nuclei were stained with DAPI. Bars = 10 µm.

Cell migration is controlled by the Rho GTPase RAC1 that, regulating actin cytoskeleton, promotes lamellipodia extension and ruffle formation^19,28–30^. Therefore, we analyzed by western blotting RAC1 protein amount in MDA-MB-231 overexpressing HA-V1G1. The analysis showed a decrease in RAC1 abundance in cells overexpressing HA-V1G1 (Fig. 4a). Therefore, we decided to analyze the levels of cofilin, a downstream effector of RAC1 that controls actin dynamics^31^, and we found a decrease of cofilin protein amount in MDA-MB-231 cells upon overexpression of HA-V1G1 (Fig. 4a).

RAC1 regulates cell protrusions, cell-extracellular matrix interactions and generation of traction forces activating vinculin, a well-characterized focal adhesion protein that plays a key role in regulating cell motility^46–48^. Therefore, we analyzed cellular localization of vinculin in MDA-MB-231 overexpressing HA-V1G1. Confocal microscopy showed a distinct localization of vinculin at the cell cortex in control cells, as expected, while in HA-V1G1 overexpressing cells vinculin appeared delocalized (Fig. 4b).

All together these data suggest that V1G1 is essential for the regulation of RAC1 and, consequently, for the activation of the downstream pathway that controls cell migration.

### Role of RAB7 in breast cancer cells

**Figure 5:**
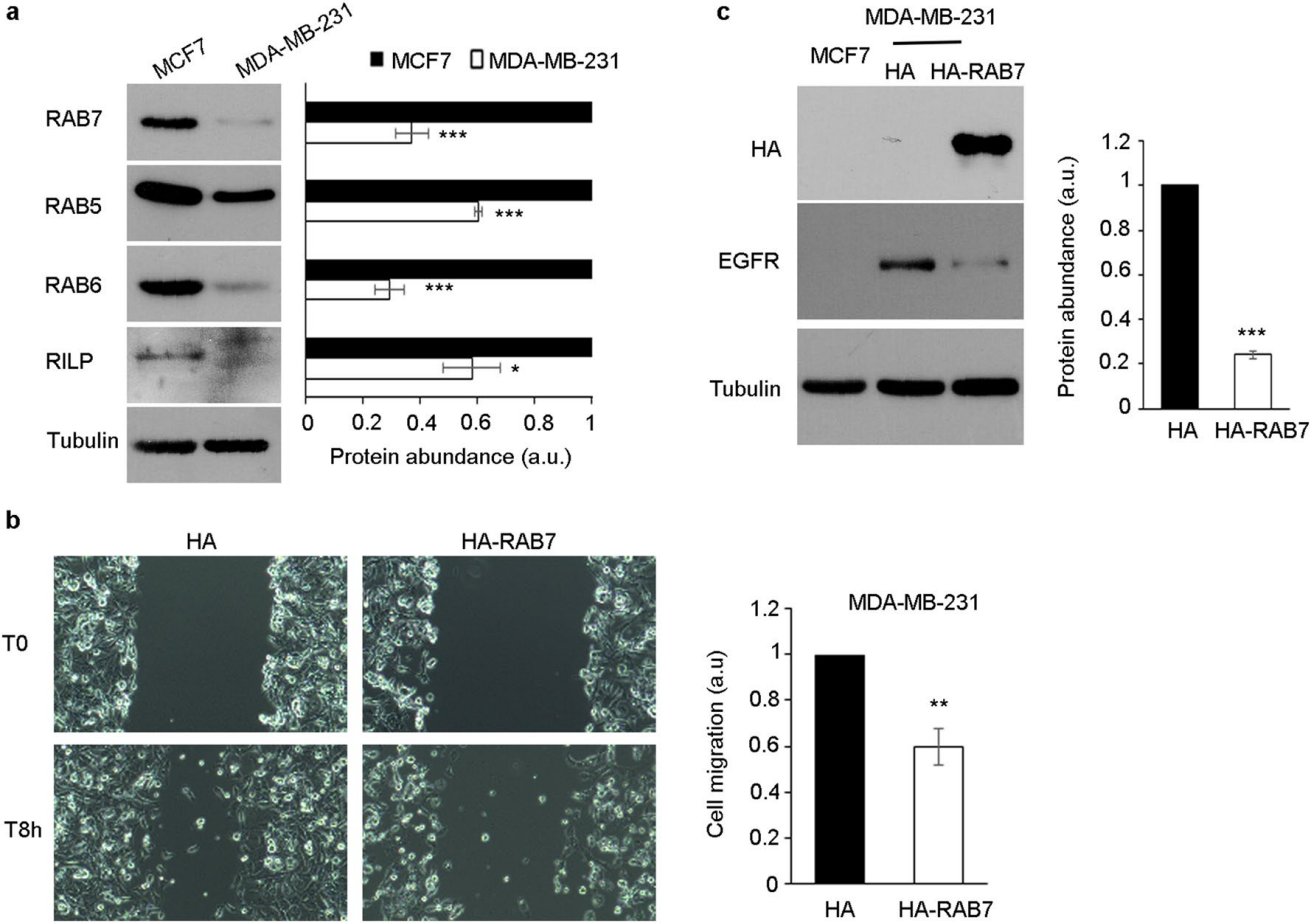
RAB7 in breast cancer cells. (**a**) Lysates of MCF7 and MDA-MB-231 cells were analyzed by Western blot using specific anti-RAB7, anti-RAB5, anti-RAB6, anti-RILP, and anti-tubulin antibodies. Intensities of bands were measured by densitometry and normalized against tubulin. (**b**) MDA-MB-231 overexpressing HA-RAB7 were imaged during wound healing assay at initial time point T0 and 8 h after the scratch. Cell migration was measured as the ratio between the closed area of the wound in control (HA) and HA-RAB7 overexpressing cells. (**c**) MDA-MB-231 overexpressing HA-RAB7 were lysed and analyzed by Western blot using specific anti-HA, anti-EGFR, and anti-tubulin antibodies. Intensities of bands were measured by densitometry and normalized against tubulin. Data represent the mean ± s.e.m. of at least three experiments. Student’s t-test. *p ≤ 0.05, **p ≤ 0.01, ***p ≤ 0.001.

V1G1 stability and localization on late endosomes and lysosomes are strongly regulated by RILP and RAB7^7,9^. The functional interaction between V1G1 and RILP/RAB7 promotes the intravesicular acidification required for maturation of endosomes and lysosomes^2,3^. Interestingly, RILP controls cell migration in MDA-MB-231 cells, and, in these cells, its expression is lower compared with the less-invasive MCF7 cell line (Fig. 5a and^49^). Thus, having demonstrated a key role of V1G1 in controlling breast cancer cell migration in vitro, we decided to analyze the role of RAB7. First, we investigated abundance of RAB7 in these cells. The analysis showed a strong decrease of RAB7 levels in MDA-MB-231 compared to MCF7 cells (Fig. 5a), associated to a decrease of the RAB7 transcript (Fig. 1b)^50^.

In addition, also in other TNB cell lines (BT-549 and Hs578T) we observed a strong decrease of RAB7 protein abundance (Fig. 1d). These data suggest that the balance of these factors is strongly regulated, as possibly the functional interaction between V1G1, RILP and RAB7 is required to control cell migration, and therefore invasiveness of breast cancer cells.

In order to evaluate whether other RAB proteins are differentially expressed in breast cancer cells, we analyzed the protein amount of RAB5, a protein involved in early steps of endocytosis, and RAB6, a protein involved in the transport from the Golgi to the endoplasmic reticulum and in exocytosis^51,52^. In MDA-MB-231 cells the levels of RAB5 and RAB6 proteins decreased compared to MCF7 cells (Fig. 5a). These data suggest an alteration of trafficking in highly invasive compared to poorly invasive breast cancer cells.

As V1G1, similarly to RILP^49,53^, drives cell migration, we analyzed the role of RAB7 in breast cancer cell migration. The wound healing assay in MDA-MB-231 cells showed that overexpression of HA-RAB7 reduced cell migration in vitro (Fig. 5b). In addition, similarly to V1G1, overexpression of HA-RAB7 reduced the amount of EGFR (Fig. 5c).

Altogether, these data indicate that the complex V1G1/RILP/RAB7 is crucial for the regulation of cell motility in vitro in breast cancer cells, suggesting that downregulation of these three factors is required for cancer progression and, in particular, for cell migration in breast cancer.

### V1G1 controls lysosome biogenesis

**Figure 6:**
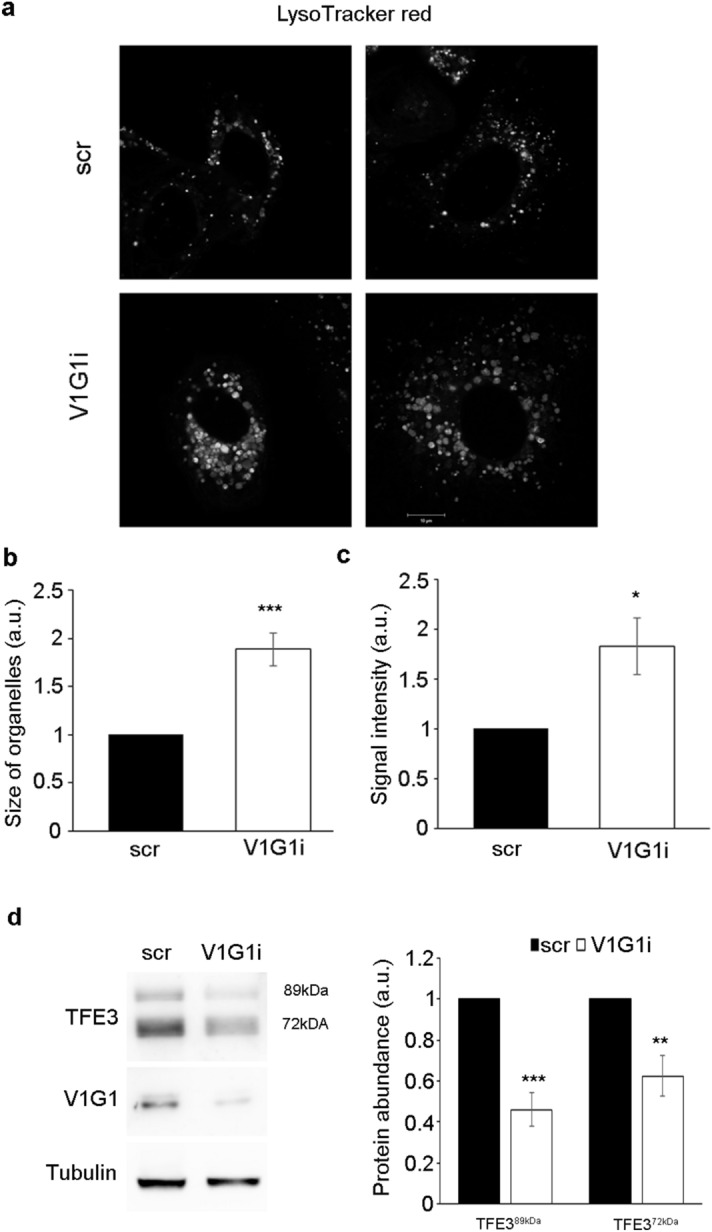
V1G1 modulates late endocytic compartments in MCF7 cells. (**a**) V1G1-depleted MCF7 cells (V1G1i) were labeled with Lysotracker Red and analyzed by confocal microscopy, scale bar: 10 µm. (**b**) The size of organelles was quantified by ImageJ software. Data represent the mean ± s.e.m. (at least 4 independent experiments, ≥ 15 cells /sample for each experiment) ***p ≤ 0.001. (**c**) Lysotracker Red intensity was quantified by ImageJ software and Corrected Total Cell Fluorescence was calculated. Data represent the mean ± s.e.m. (at least 4 independent experiments, ≥ 15 cells/sample for each experiment) *p ≤ 0.01. (**d**) Relative protein abundance of the two active isoforms of TFE3 and V1G1 was assessed by western blotting, quantified by densitometry and normalizing against tubulin. Data represent the mean ± s.e.m of at least three independent experiments (*p ≤ 0.01, **p ≤ 0.01, ***p ≤ 0.001).

V-ATPase controls maturation of late endosomes and lysosomes^3^. In order to establish the role of V1G1 in this process, we monitored intracellular distribution of lysosomes in V1G1 depleted MCF7 cells using a specific vital dye, LysoTracker DND-99 that stains acidic organelles in live imaging microscopy. As shown in Fig. 6a, V1G1 depleted MCF7 displayed a significant increase of the dimension of acidic compartments stained with LysoTracker DND-99 (Fig. 6b) and intensity quantification revealed an increase of about twofold in V1G1 depleted compared to control cells (Fig. 6c). These data demonstrate that acidic compartments are affected by V1G1 depletion. Thus, we decided to analyze the protein abundance of TFE3, a master regulator of lysosome biogenesis^54^. Western blot analysis revealed a strong reduction of TFE3 expression in V1G1 depleted cells (Fig. 6d) suggesting that V1G1 plays a critical role in lysosomal biogenesis.

## Discussion

V-ATPase is a multi-subunit complex composed by several subunits that control activation and regulation of the pump in different location^1–3^. Multiple isoforms exist for many subunits of the pump and these isoforms are ubiquitous or cell- or organelle-specific^3^. Notably, abnormal expression of specific isoforms of the pump correlates with cancer cell aggressiveness^1–3,11–15,55^. Here, we showed that expression of V1G1, a component of the peripheral stator of the pump, is strongly decreased in TNB breast cancer cells compared to breast cancer cells of other subtypes (Fig. 1d). This finding was confirmed by analysis of V1G1 expression using the UALCAN database (Fig. 1c). This downregulation, in TNB breast cancer cells, is associated to the previously shown increase in V1C1 protein amount (Fig. 1a)^38^, suggesting that V1G1 negatively regulates breast cancer migration in vitro. Indeed, we showed a correlation between V1G1 expression and cell migration in vitro as in TNB cell lines low levels of V1G1 are associated to an increase of cell migration compared to luminal A subtype MCF7 (Fig. 1d, e). As the G subunit of the V1 domain has three isoforms^3^, it will be interesting in the future to evaluate expression of the other subunits because in the G2-null mouse brain a switch between the different G isoforms has been observed as a compensatory mechanism^56^.

Interestingly, in TNB cell lines we observed also a decrease of RAB7 protein abundance (Figs. 1d, 5a). RAB7 is a key regulator of endocytosis that controls, together with RILP, protein stability of V1G1 required for the recruitment and activation of the pump on endolysosomal membranes^7,8^. Silencing of V1G1 in MCF7 cells affects lysosomal morphology as lysosomes looks larger (Fig. 6a, b) and, notably, increased size of lysosomes is often associated to pathological conditions^57^. Moreover, the decrease in the amount of TFE3 (Fig. 6d), a master regulator of lysosomal biogenesis^54^, suggests that V1G1 controls lysosome biogenesis. Accordingly, overexpression or silencing of V1G1 in HeLa cells affects cathepsin D maturation^7^ and EGF-induced EGFR degradation (Fig. 3a, b).

**Figure 7:**
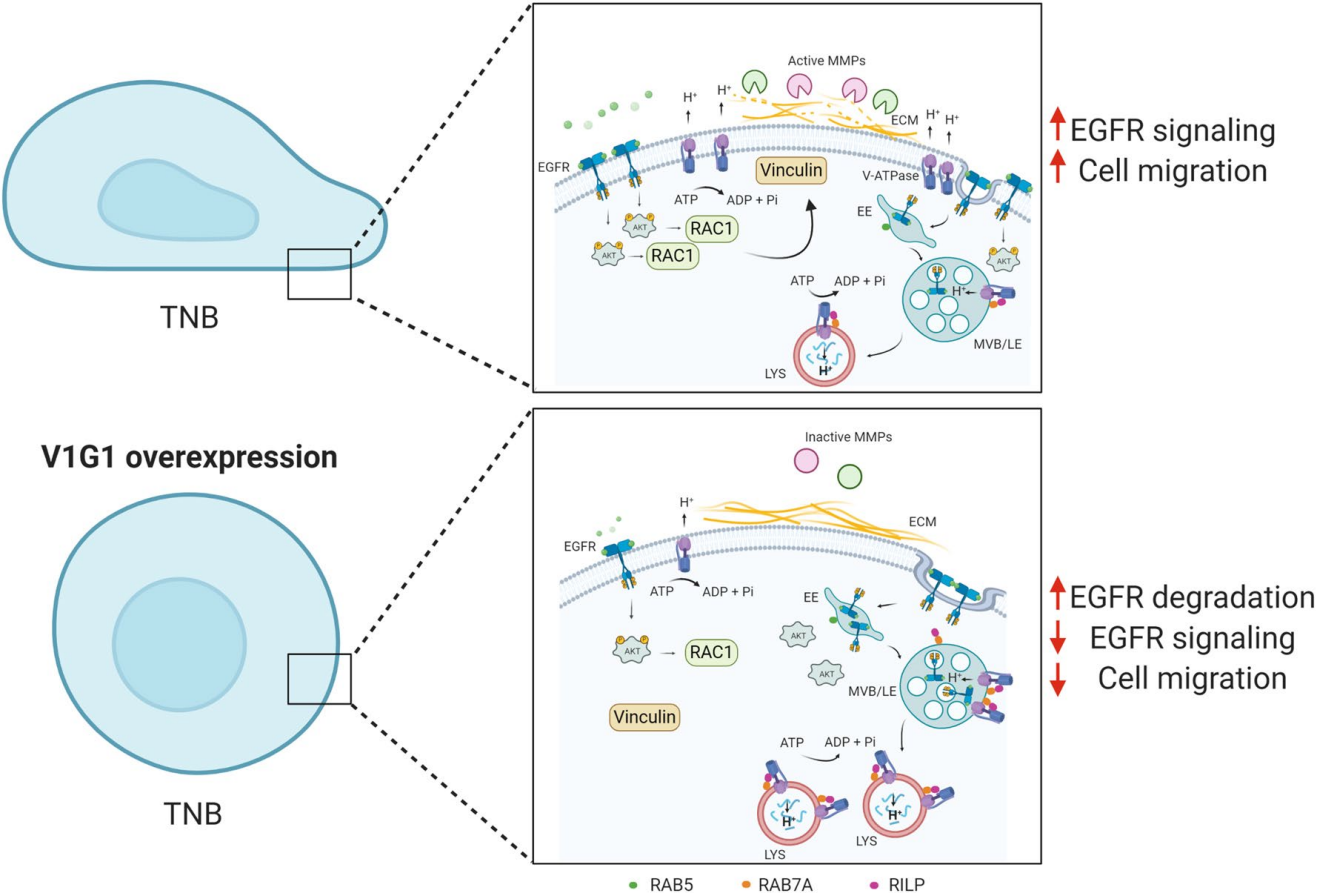
Proposed molecular model. In TNB breast cancer cell lines, overexpression of V1G1 affects cell migration in vitro by modulating EGFR stability and signaling that control activation of RAC1 and vinculin assembly. Similarly, overexpression of RAB7 affects EGFR degradation but also positioning of V-ATPase on endocytic organelles through RILP. In addition, overexpression of V1G1 reduces activation of MMPs that degrade extracellular matrix favoring cell migration in vivo. *ECM* extra-cellular matrix, *EE* early endosome, *MVB* multivesicular bodies, *LE* late endosome, *LYS* lysosome. Created with Biorender.com.

Inappropriate activation of EGFR in cancer drives tumorigenesis and is associated to poor prognosis^22^. Increased expression and/or gene amplification of EGFR have been observed in many human cancers including triple negative breast cancer and, accordingly, MDA-MB-231 cells express high levels of EGFR compared to MCF7 cells that belong to the luminal A subtype (Fig. 3c)^26,58,59^. To mimic MCF7 condition, we overexpressed V1G1 or RAB7 in MDA-MB-231 and Hs578T cells and we observed a decrease of EGFR amount associated with a decrease in its activation (Figs. 3c, e, 5c). RAB7 and RILP control EGFR downregulation^6,60–63^, possibly also through the recruitment of the V-ATPase proton pump on the membranes of endosomes and lysosomes, mediated by the V1G1 subunit, required for their maturation^7^. Thus, we suppose that overexpression of V1G1 and RAB7, in MDA-MB-231, promotes recruitment of the pump on endocytic organelles negatively affecting the recruitment of the pump on the plasma membrane (Fig. 7). Also, upon overexpression of HA-V1G1 or HA-RAB7, TNB cells showed a decrease of EGFR and of LDLR (Figs. 3c, d, 5c) and a simultaneous decrease of cell migration (Figs. 2b, c, 5b), similarly to what happens upon RILP overexpression^49^. On the contrary, V1G1 depletion in MCF7 cells increased cell migration in vitro (Fig. 2a), as observed upon RILP depletion^49,53^. In light of these data, it will be interesting in the future to validate the role of V1G1 in tumor invasiveness in vivo.

The signaling of EGF activates RAC1 to control cytoskeletal reorganization, a crucial event in the metastatic cascade^19^. Overexpression of RAC1 correlates with poor prognosis in breast cancer cells^27^, and the pharmacologic reduction or knockdown of V-ATPase activity significantly reduce migration of invasive tumor cells in vitro preventing activation of EGFR and RAC1^16^. In MDA-MB-231 cells we observed a decrease in RAB7 abundance, but we have previously associated RAB7 depletion to a decrease in cell migration combined to alterations in β1-integrin activation, distribution and trafficking and decreased RAC1 activation^64^. We suppose that, in MDA-MB-231 cells, overexpression of RAC1 could bypass the RAB7-dependent negative regulation of cell motility. In fact, in these cells overexpression of V1G1 reduces cell migration (Fig. 2b), RAC1 total protein amount (Fig. 4a) and, at the same time, we observed a decrease of cofilin, a key regulator of actin dynamics that promotes cell migration^31^ (Fig. 4a).

RAC1 regulates the activation of vinculin, a well-characterized focal adhesion protein involved in many steps of cell migration^46,48^. In triple negative breast cancer cells reduction of tumorigenic phenotypes is accompanied by reduced expression of RAC1, alpha-actinin, vinculin and FAK^65^ and silencing of vinculin reduces cell viability and decreases tumor volume in animal models^66^. The decrease in vinculin assembly observed in MDA-MB-231 cells overexpressing V1G1 is associated to a decrease in RAC1 protein amount (Fig. 4a). Therefore, the decrease of cell migration observed in these cells (Fig. 2b) could be associated to a reduced assembly of vinculin at the leading edge (Fig. 4b). Altogether, these data showed that HA-V1G1 overexpression in MDA-MB-231 cells downregulates EGFR signaling that, through the activation of RAC1, controls cell migration (Fig. 7).

The promoting effect of V-ATPase on cancer invasion is ensured also by maintaining acidic extracellular microenvironment required to increase secretion and activation of degradative enzymes such as matrix metalloproteinases that, by remodeling the extracellular environment, promote cell migration and invasion^37^. MMP-2 and MMP-9 are highly expressed in malignant tumors and positively correlate with an aggressive malignant phenotype and poor outcome in breast cancer patients^34,67^. Accordingly, we observed that V1G1 overexpression decreases the activation of secreted MMP-2 and MMP-9 (Fig. 2d, e), thus confirming a role of this subunit in processes related to cell migration.

## Conclusion

Altogether these data indicate that V1G1 has a key role in the regulation of several processes that promote the invasive phenotype of breast cancer cells and may be a novel cell migration suppressor factor. In particular, V1G1, controlling EGFR stability, modulates EGFR downstream signaling pathways required for the cell migration (Fig. 7).

## Methods

### Cells and reagents

MCF7, MDA-MB-231, MDA-MB-415, MDA-MB-453, MDA-MB-468, BT-549, Hs578T and HeLa cells were grown in Dulbecco's Modified Eagle's medium (DMEM), HCC1428, T47D, ZR75-1 were grown in RPMI in a 5% CO_2_ incubator at 37 °C. Media were supplemented with 10% Fetal bovine serum (FBS), 2 mM L-glutamine, 100 U/ml penicillin and 10 mg/ml streptomycin. Cell lines used were from ATCC (Manassas, VA, USA). Cells were periodically checked to ensure that they were free from mycoplasma infections. The reagents for tissue culture were from Sigma-Aldrich (St-Louis, MO, USA) or Gibco (Gibco, Grand Island, NY, USA). The ATP-competitive pan-Akt inhibitor, GDC-0068, was from Cayman Chemical Company (Monmouth Junction, NJ, USA).

### Transfection and RNA interference

Transfection was performed using Lipofectamine LTX Reagent with PLUS Reagent (Invitrogen, Carlsbad, CA, USA) or Metafectene Pro from Biontex (München, DE) according to the manufacturer’s instructions. When not indicated cells were processed 24 h after the transfection. HA-V1G1 and HA-RAB7 constructs used in this study have been described previously^7,63^.

The pCDNA3_2xHA was generated by cloning the fragment of 2xHA 5′ TAC CCA TAC GAT GTT CCG GAT TAC GCT TAC CCA TAC GAT GTT CCG GAT TAC GCT 3′, flanked by a sequence containing the site for KpnI enzyme, into the vector pCDNA3 (Invitrogen).

MCF7 and HeLa cells were silenced with small interfering RNAs (siRNAs) purchased from Eurofins Genomics (Ebersberg, Germany). We used the following oligonucleotides: control RNA, sense 5′-ACUUCGAGCGUGCAUGGCUTT-3′ and antisense 5′-AGCCAUGCACGCUCGAAGUTT-3′; siRNA-V1G1, sense 5′-AGAAGAAGCUCAGGCUGAATT-3′ and antisense 5′-UUCTGCCTGAGCUUCUUCUTT-3′;

For silencing, HeLa cells were transfected using Oligofectamine Transfection Reagent (Invitrogen) according to the manufacturer’s instructions for 72 h, re-plated and left 48 h before performing further experiments. MCF7 cells were transfected using Metafectene SI (Biontex) according to the manufacturer’s instructions and were processed 48 h after the transfection.

### Standard RNA procedures and quantitative real-time PCR

RNA extraction, retrotranscription and quantitative real-time PCR have been previously described^7^.

The primers used were: GAPDH *Forward*: 5′GGTGGTCTCCTCTGACTTCAACA-3′ *Reverse*: 5′-GTTGCTGTAGCCAAATTCGTTGT-3′; V1G1 *Forward:* 5′-GCCGAGAAGGTGTCCGAGGCCCG-3′ *Reverse*: 5′ -GCGGTACTGTTCAATTTCAGCC-3′; RAB7 *Forward*: 5′-CACAATAGGAGCTGACTTTCTGACC-3′, *Reverse*: 5′-GTTCCTGTCCTGCTGTGTCCCATATC-3′purchased from Eurofins Genomics. The PCR program was as follows: 1 cycle 3 min at 94 °C; 35 cycles 30 s at 94 °C, 30 s at 60 °C, 30 s at 72 °C; 1 cycle 6 min at 75 °C. The specificity of PCR products was checked by performing a melting-curve test. The relative expression level was calculated using the comparative C_T_ method and expressed as a “fold change”, as described previously^60^.

### Western blotting

Cells were lysed with RIPA or Laemmli buffer and processed for Western blot analysis as previously described^7,60^. Signal was captured on a film or using Bio-Rad ChemiDoc MP Imaging Systems (Hercules, CA, USA). Densitometric analysis was performed using NIH ImageJ (Bethesda, MD, USA) or Image Lab software (Bio-Rad). Rabbit anti-RILP antibody was previously described^5^. Commercial antibodies used are listed in Table 1. HRP-conjugated secondary antibodies were from Invitrogen or Santa Cruz Biotechnology (Santa Cruz, CA, USA).

**Table 1:** Antibodies panel. List of antibodies used in this study.

Antibody	Species	Dilution	Application	Cat. No	Company
Anti-AKT	Rabbit	1:1000	WB	4691	Cell Signaling Technology (Leiden, The Netherlands)
Anti-pakt	Rabbit	1:200	WB	sc-7985-R	Santa Cruz Biotechnology
Anti-cofilin	Rabbit	1:200	WB	sc-33779	Santa Cruz Biotechnology
Anti-EGFR	Sheep	1:1000	WB	20-ES04	Fitzgerald ((North Acton, MS, USA)
Anti-Erk	Rabbit	1:200	WB	sc-93	Santa Cruz Biotechnology (Dallas, TX, USA)
Anti-perk	Mouse	1:200	WB	sc-7383	Santa Cruz Biotechnology
Anti-HA	Rabbit	1:500	WB	sc-805	Santa Cruz Biotechnology
Anti-HA	Mouse	1:500	WB	sc-7392	Santa Cruz Biotechnology
Anti-HA	Rabbit	1:2000	IF	ab9110	Abcam (Cambridge, UK)
Anti-LDLR	Rabbit	1:1000	WB	PAB8804	Abnova (Taipei, Taiwan)
Anti-MMP-2	Rabbit	1:1000	WB	4022	Cell Signaling Technology
Anti-RAB5	Rabbit	1:200	WB	sc-309	Santa Cruz Biotechnology
Anti-RAB6	Mouse	1:500	WB	sc-81913	Santa Cruz Biotechnology
Anti-RAB7	Mouse	1:500	WB	sc-376362	Santa Cruz Biotechnology
Anti-RAC1	Mouse	1:600	WB	ARC03	Cytoskeleton (Denver, CO, USA)
Anti-TFE3	Rabbit	1:500	WB	HPA023881	Sigma-Aldrich
Anti-tubulin	Mouse	1:10,000	WB	T5168	Sigma-Aldrich
Anti-V0D1	Mouse	1:100	WB	sc-81887	Santa Cruz Biotechnology
Anti-V1C1	Rabbit	1:1000	WB	361-375	Sigma-Aldrich
Anti-V1G1	Chicken	1:1000	WB	ab15853	Sigma-Aldrich
Anti-V1G1	Mouse	1:100	WB	sc-25333	Santa Cruz Biotechnology
Anti-vinculin	Mouse	1:50	IF	9131	Sigma-Aldrich

*WB* western blotting, *IF* immunofluorescence.

### Wound-healing assay

The wound healing assay was performed as previously described^60,68^. Cells were imaged in at least 3 fields at the moment of the scratch (T0) and after 8 h or 24 h. We measured the wound area with the software ImageJ for each time point and we calculated the closed area of the wound for each sample as the difference between the wound area at T0 and at T8h or T24h. We set to 1 the closed area in the control cells and we measured cell migration as the ratio between the closed area of the wound in control cells and HA-V1G1 overexpressing or V1G1 silencing cells. When indicated, we performed the wound healing assay on confluent monolayer of MDA-MB-231 cells treated for 24 h with the inhibitor GDC-0068 (1 µM) or with DMSO as control. Cells, in medium supplemented with the inhibitor, were imaged at the moment of the scratch (T0) and after 8 h.

### Gelatin zymography

Gelatin zymography was performed with 3ug of each sample, as previously described^60^. Briefly, secreted proteins were separated under denaturing but non reducing conditions through a polyacrylamide gel containing gelatin, a synthetic substrate of the gelatinases. Coomassie Blue staining allowed visualization of the clear region (the gelatin digested zone) on a uniform blue background of undegraded substrate^44^. Using the NIH ImageJ software, the clear region on a uniform blue background was quantify for each sample.

### EGFR degradation assay

To measure EGFR degradation cells were treated and processed as previously described^60^.

### Confocal immunofluorescence microscopy and live imaging

MDA-MB-231 cells were fixed for 15 min with 3% paraformaldehyde at room temperature, permeabilized for 5 min with 0.1% Triton X-100 and then processed as previously described^60^. Primary antibodies used are described in Table 1, while secondary antibodies conjugated with fluorophores were from Invitrogen. For live microscopy, V1G1 depleted and control MCF7 cells were seeded into microscopy chambers (8 well µ-slide, Ibidi GmbH, Martinsried, Germany) and 48 h after silencing were incubated with 0.5 µM LysoTracker Red DND-99 (Thermo Fisher Scientific, Carlsbad, CA, USA) for 30 min at 37 °C. After 3 washes in PBS, L-15 medium (Leibowitz medium without phenol red, Gibco) was added and the cells were imaged by a confocal laser scanning microscope (CLSM) (Zeiss, LSM 700, Germany). Images were acquired using ZEN Black Edition 2011 software (Zeiss, Jena, Germany). ImageJ software was used to measure the size of organelles and the intensity of fluorescence calculated as CTCF (Corrected Total Cell Fluorescence) with the following formula: CTCF = Integrated Density − (Area of selected cell × Mean fluorescence of background readings). Measures were obtained by analyzing at least 15–20 cells/sample in four independent experiments.

### Analysis of V1G1 expression level between breast cancer and normal tissues

To analyze TCGA level RNA-seq of V1G1 in various breast cancer subclasses we used UALCAN (http://ualcan.path.uab.edu)^39^. With the Human Protein Atlas (www.proteinatlas.org), we detected the V1G1 protein expression among normal and cancer tissues^40^.

### Statistical analysis

All experiments were conducted at least 3 times. Results are expressed as mean value ± SEM. Data were statistically analysed using Student's t-test. Comparisons between multiple groups were made by one-way ANOVA followed by Dunnett’s multiple comparisons test using GraphPad Prism (*p ≤ 0.05, **p ≤ 0.01 and ***p ≤ 0.001).

## Supplementary information

Supplementary information.

## Data Availability

All data are available from the first author on request.
