# Supplementary material for: Role of the V1G1 subunit of V-ATPase in breast cancer cell migration
Source: Sci Rep. 2021 Feb 25;11:4615. doi: 10.1038/s41598-021-84222-9 (PMC7907067; doi:10.1038/s41598-021-84222-9)

**Supplementary Information for**

**Role of the V1G1 subunit of****V-ATPase in breast cancer cell migration**

Maria De Luca*, Roberta Romano and Cecilia Bucci*

Department of Biological and Environmental Sciences and Technologies, University of Salento, Via Provinciale Lecce-Monteroni n. 165, 73100 Lecce, Italy.

***Corresponding authors:**

MDL, [maria.deluca@unisalento.it](mailto:maria.deluca@unisalento.it); CB, [cecilia.bucci@unisalento.it](mailto:cecilia.bucci@unisalento.it)

**Supplementary Figure 1: The expression of V1G1 in normal and cancer tissues. A-B)** Overview of V1G1 protein levels in normal and cancer tissues. Data derived from the Human Protein Atlas database.


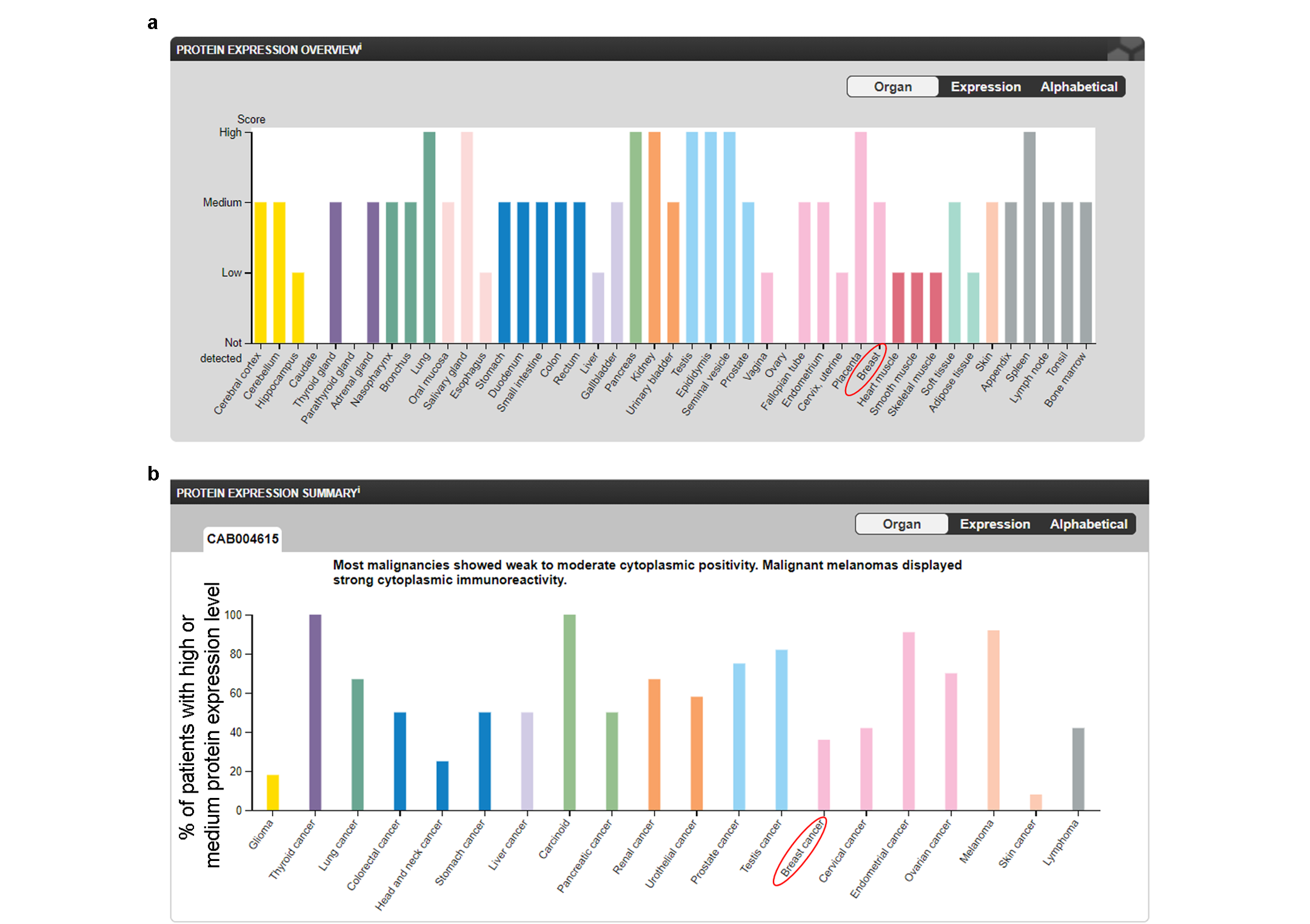


**Full-length gels / blots**


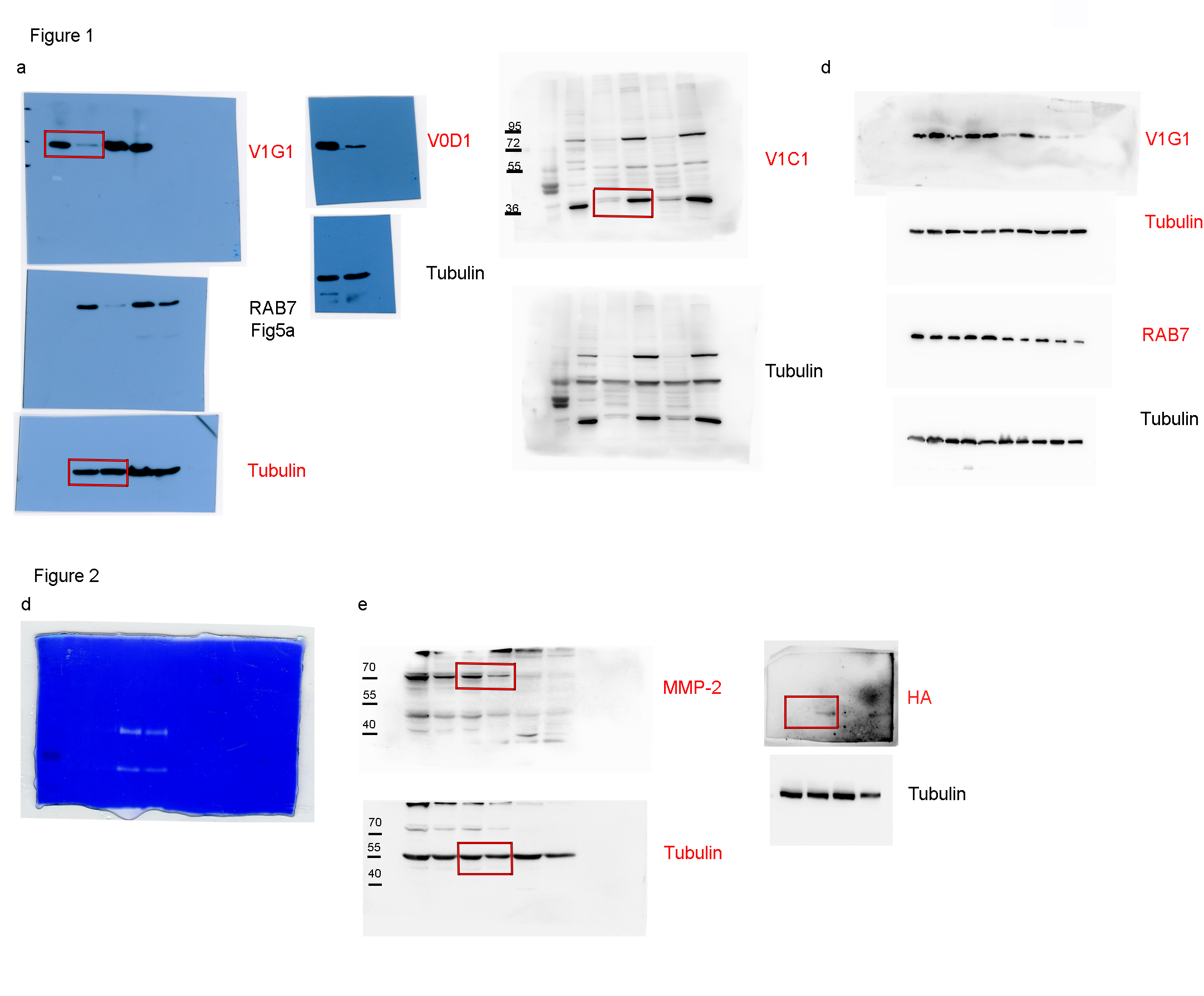


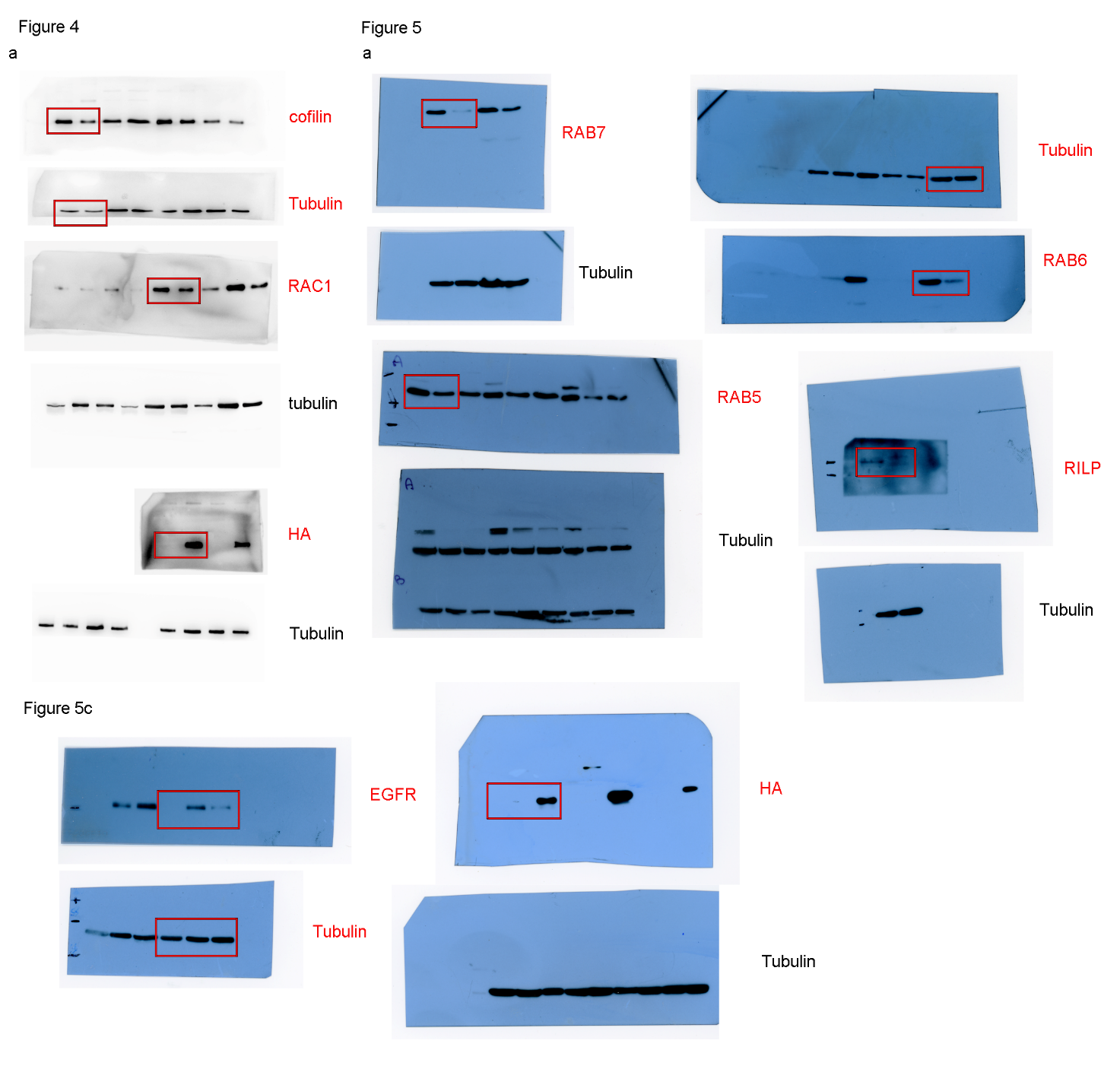


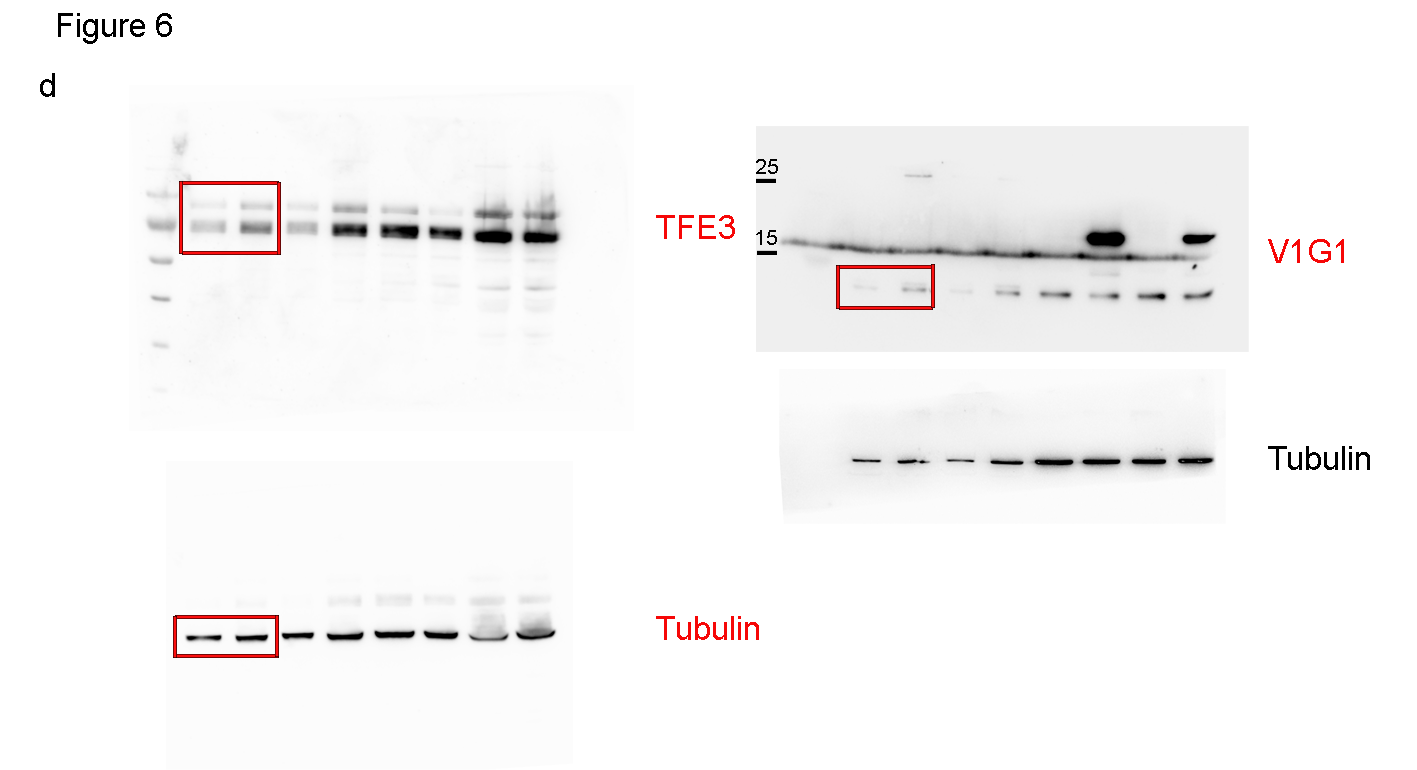

Supplement: Supplementary file 1 — Supplementary information. [file 41598_2021_84222_MOESM1_ESM.docx]
